# Peripartum Cardiomyopathy: Management Strategies for Pregnancy Termination

**DOI:** 10.1089/whr.2020.0078

**Published:** 2020-10-08

**Authors:** Ashley M. Darlington, Jonah D. Fleisher, Joan E. Briller

**Affiliations:** ^1^ Department of Medicine, University of Illinois at Chicago, Chicago, Illinois, USA.; ^2^ Department of Obstetrics and Gynecology, University of Illinois at Chicago, Chicago, Illinois, USA.; ^3^ Division of Cardiology, Department of Medicine, University of Illinois at Chicago, Chicago, Illinois, USA.

**Keywords:** case reports, perioperative management, peripartum cardiomyopathy, pregnancy termination

## Abstract

Some women have underlying cardiovascular disease that leads to increased morbidity and mortality with pregnancy. These women may choose to terminate a pregnancy rather than face this increased risk. The optimal approach for pregnancy termination in women with cardiomyopathy is not well defined. We present two women with peripartum cardiomyopathy, both modified World Health Organization (mWHO) class IV and with elevated Cardiac Disease in Pregnancy (CARPREG II) pregnancy risk stratification scores who are at the highest risk for pregnancy continuation. Both underwent induced abortion, although the procedure was performed in very different settings. These cases illustrate factors that influence the mode and setting of pregnancy termination performance.

## Introduction

Cardiovascular disease (CVD) has emerged as a leading cause of maternal mortality. It is estimated that 17.2% of women aged 20–39 years in the United States have underlying CVD that can complicate pregnancies and lead to increased morbidity and mortality.^1–3^ Although women with CVD at the highest risk ideally should prevent unwanted pregnancies, many pregnancies are unplanned or occur due to contraceptive failures.^4^ Under these circumstances, women may choose to end their pregnancies because of, or in the context of, cardiomyopathy and the elevated pregnancy-related morbidity and mortality posed. We present two women with peripartum cardiomyopathy (PPCM) who underwent first-trimester pregnancy termination in different settings to illustrate factors to be considered in the counseling and management patients with PPCM during and after induced abortion.

## Case 1

### Presentation

A 23-year-old G1P1 woman with no prior cardiac history and an uncomplicated pregnancy presented 3 months postpartum with dyspnea on exertion, orthopnea, and a positive pregnancy test. She was diagnosed with a new first-trimester pregnancy after a short interpregnancy interval.

### Investigations

**FIG. 1.**
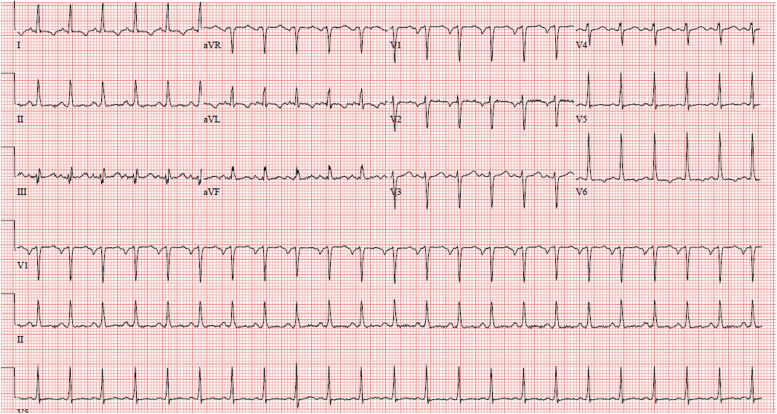
Electrocardiogram at presentation for case 1 shows sinus tachycardia with a short PR interval and ST segment and T wave abnormalities in the lateral leads.

Blood pressure (BP) was 95/70, and pulse was 139 bpm. Physical examination revealed elevated jugular venous pressure (JVP), S3, and peripheral edema. Twelve-lead EKG showed sinus tachycardia with short PR, ST, and T wave abnormality lateral leads (Fig. 1). Laboratory tests demonstrated troponin 0.09 ng/mL (normal ≤0.04 ng/mL) and brain natriuretic peptide 1014 pg/mL (normal ≤100 pg/mL). Transthoracic echocardiogram (TTE) revealed left ventricular (LV) enlargement with severely reduced left ventricular ejection fraction (LVEF), <20%, diastolic parameters were compatible with grade 3 diastolic dysfunction with increased LV end diastolic pressure, mild right ventricular enlargement with right ventricular dysfunction and moderate to severe mitral insufficiency. CT pulmonary embolism protocol showed cardiomegaly with pleural effusions but was negative for pulmonary embolism. Findings were consistent with a diagnosis of PPCM. Symptoms were compatible with New York Heart Association (NYHA) class IV heart failure at presentation. Transvaginal ultrasound confirmed a 7-week intrauterine pregnancy.

### Management

Her heart failure symptoms improved with diuresis. She was started on guideline-directed medical therapy, initially with hydralazine/nitrates and subsequently with an ACE inhibitor and beta blockade. Potential use of bromocriptine was discussed with the patient who declined. Estimated modified World Health Organization (mWHO) pregnancy class was IV due to LV dysfunction and Cardiac Disease in Pregnancy (CARPREG II) score was 6 given heart failure symptoms, ventricular dysfunction, and no prior cardiac intervention.^2,3^ The patient's high risk of mortality and morbidity with a continued pregnancy were addressed with the patient and she made the decision to terminate her pregnancy. After initial cardiac stabilization, the patient underwent induced abortion at 8 weeks' gestation through vacuum aspiration (dilation and curettage) in the surgical operating room with telemetry monitoring. The time from presentation to induced abortion was 5 days. She also requested and received a levonorgestrel intrauterine device (Mirena^®^; Bayer, Whippany, NJ) for contraception. During her hospitalization, she was noted to have nonsustained ventricular tachycardia and discharged with a prophylactic LifeVest^®^ (ZOLL Medical Corporation, Pittsburgh, PA) and plans for anticoagulation with warfarin although she did not follow up with anticoagulation clinic.

### Outcomes

She had no immediate postoperative complications, but was rehospitalized 1 month later with cardiogenic shock refractory to medical therapies and was transferred to a nearby transplant center for advanced heart failure interventions. Subsequent course has included placement of an intracardiac defibrillator and amiodarone therapy secondary to ventricular tachycardia and placement of an LV assist device with improved heart failure symptoms. Current NYHA class is I–II.

## Case 2

### Presentation

**FIG. 2.**
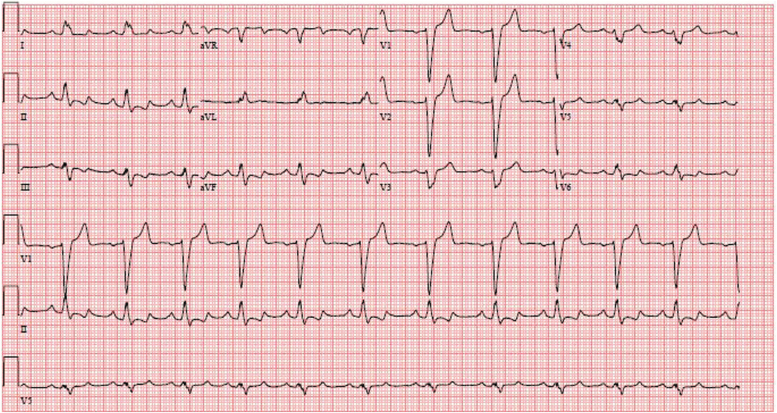
Electrocardiogram at presentation for case 2 shows sinus rhythm with left bundle branch block.

A 34-year-old G3P2012 woman with a history of PPCM diagnosed 1 year previously presented with a pregnancy at 5-weeks' gestation. At time of initial PPCM diagnosis, BP was 130/97 and pulse was 98. Physical examination demonstrated normal JVP with negative hepatojugular reflex, no peripheral or pulmonary edema, and normal heart sounds without S3. EKG showed sinus rhythm with left bundle branch block (Fig. 2). TTE showed severe LV enlargement with LVEF 25%–30%, compatible with diagnosis of PPCM. Initial brain natriuretic peptide (BNP) available was 325 pg/mL (normal ≤100 pg/mL). She was started on guideline-directed medical therapy with beta-blocker and ace-inhibitor therapy but ultimately transitioned to a neprilysin inhibitor with symptom resolution. Clinically, and by exercise testing, she was NYHA class 1; however, her ejection fraction as measured by TTE did not improve. Prior evaluation by electrophysiology recommended that she did not require placement of an implantable cardiac defibrillator, nor would she benefit from placement of a resynchronization device, since her LV function by cardiac magnetic resonance appeared better than by echocardiography and she had no history of ventricular arrhythmias.

### Investigations

At time of evaluation by family planning, BP 125/76 and pulse was 66. Physical examination reported normal heart sounds without S3 and no peripheral or pulmonary edema. N,T pro-BNP was minimally elevated at 194 pg/mL (normal ≤124 pg/mL). Transvaginal ultrasound was consistent with pregnancy at <6 weeks' gestation.

### Management

Current mWHO risk was determined to be class IV in view of her history of PPCM with residual LV dysfunction and CARPREG II score 5 (for history of heart failure and current LV dysfunction). Usual heart failure therapy was continued and a vacuum aspiration was performed for termination in the outpatient gynecology clinic on the day she was evaluated by family planning clinic.

### Outcomes

There were no postprocedural complications after the pregnancy termination. She planned to use condoms until her partner was able to obtain a vasectomy. One year later, her LVEF remains unchanged and she is clinically stable (NYHA class I).

## Discussion

Several indices have been developed to address likelihood of pregnancy complications in women with known CVD. The mWHO classification scheme was originally adapted from an approach to assess contraceptive safety.^5^ mWHO class integrates lesion-specific information for a variety of congenital and acquired conditions and divides them into four classes. Class I conditions, for example, are low risk with a complication rate similar to the general population. Class IV patients are at the highest risk and pregnancy considered contraindicated. This approach is endorsed by the European Society of Cardiology guidelines for management of CVD in pregnancy and is utilized widely.^2^ The CARPREG, originally published >20 years ago, also included women with both congenital and acquired heart disease. Pregnancy risk is stratified by functional class, and presence of cyanosis, arrhythmias, prior cardiac events, left heart obstruction (aortic or mitral stenosis or hypertrophic cardiomyopathy) or ejection fraction ≤40%.^6,7^ CARPREG II is a prospective multicenter analysis of almost 2000 pregnancies in women, which updated this tool by adding four lesion-specific predictors (presence of a mechanical prosthetic heart valve, high-risk aortopathy, pulmonary hypertension, or coronary artery disease) and a delivery predictor (gestational age at the time of initial pregnancy assessment).^3^ Higher scores with either approach are associated with increased morbidity and mortality with pregnancy. Both frameworks provide information useful for counseling women about risk of continuing a pregnancy.

Women with a history of PPCM and persistent LV dysfunction face a high risk of worsening LV function in subsequent pregnancies and are often taking teratogenic medications that must be discontinued during pregnancy potentially contributing to pregnancy risk.^8^ Both women were mWHO class IV and had elevated CARPREG II scores. These are associated with an extremely high risk of maternal mortality and morbidity, with an anticipated cardiac event rate of 40%–100%.^2,3^ When pregnancy poses such high risks to a woman, recommendations typically include avoidance of pregnancy and use of highly effective contraceptive methods, but pregnancies do still occur. After considering their options, both pregnant women reported in this study chose induced abortion, although the procedure was performed in very different settings.

Both medical and surgical methods for induced abortion are safe and effective.^9^ First-trimester surgical abortion efficacy at ending a pregnancy is as high as 99.8%, with a major complication rate of <0.1%. Anesthesia may pose a higher risk to those with CVD, but the procedure is often performed using only nonsteroidal anti-inflammatory drugs and local anesthesia through paracervical block. Medical abortion up to 10 weeks' gestation is equally safe and effective in the general population,^9^ but its safety in the patient with CVD has been questioned. Physiological data have shown that misoprostol, a key medication used in medical abortion, has no effect on cardiac function, but several case reports associate this drug with serious cardiac events, including myocardial infarction, attributed to coronary spasm in women at higher risk (such as older age or other risk factors for coronary disease).^10,11^ Medical abortion carries a 2.2% risk of unanticipated (but not necessarily urgent) vacuum aspiration for cases of failed abortion or incomplete abortion. This risk is higher than the 0.6% risk of unanticipated vacuum aspiration with surgical abortion.^9^ Based on these considerations, the European Society of Cardiology has suggested performing a surgical abortion in highest-risk patients, and to limit the dose of misoprostol if it is used.^2^

Workup and optimization of the gravida seeking termination is more urgent than for many of the patients for whom the cardiologist may be consulted for perioperative planning. The risk for cardiovascular and pregnancy-related complications rises with each week of gestation, so expediting care will minimize cardiovascular and gynecological morbidity and complications. Extensive preoperative evaluation runs the risk of significant delays in performing surgery.^12^

These cases illustrate that the functional capacity and degree of compensation at pregnancy presentation can greatly affect management of pregnancy termination. In case 1, the patient presented with decompensated heart failure and poor functional capacity (NYHA class IV symptoms) that only improved to NYHA class III symptoms after stabilization. Expediting workup and stabilization in the hospital setting was critical and facilitated coordination with obstetric anesthesiology for telemetry, pain control, and preparedness for emergent interventions if adverse events arose after termination. In case 2, the woman was asymptomatic on guideline-directed medical therapy, had good functional capacity (NYHA class I), and was hemodynamically compensated on examination when she became pregnant. She desired that the procedure be performed as soon as possible for her psychological well-being without waiting for a scheduled date in the operating room. We suggest that approach to termination should be individualized based on multiple factors: gestational age, patient-specific characteristics, patient and physician preference, and especially preoperative input by a cardiologist and potentially obstetric anesthesiologist for more complex patients. Finally, these cases illustrate the importance of discussing contraception with women at the highest risk of pregnancy complications. Safe contraceptive care is available for all women with CVD.^13^

## Conclusions

Cardiologists may be called upon to care for pregnant women with CVD: for risk stratification and advice about the safety of continuing their pregnancy, for managing those risks if the goal is childbirth, and for assistance with choosing the safest method for abortion when they choose to end their pregnancy. In particular, those with PPCM will require cardiovascular expertise for perioperative management. Medical termination using misoprostol has possible cardiac risk. However, surgical termination can prompt lengthy preoperative evaluations that may delay termination to a later gestational age and, therefore, increase procedural risk. Further research is necessary to determine the optimal management of women with CVD seeking pregnancy termination. This case series was reviewed by the institutional review board at the University of Illinois at Chicago (UIC) as protocol 2019-0377 with title “Pregnancy and Cardiovascular Disease: Two case reports on management of pregnancy termination” and deemed exempt.

## Abbreviations Used

BNP: brain natriuretic pepetide
BP: blood pressure
CARPREG II: Cardiac Disease in Pregnancy Study
CT: computed tomography
CVD: cardiovascular disease
JVP: jugular venous pressure
LV: left ventricular
LVEF: left ventricular ejection fraction
NYHA: New York Heart Association
mWHO: modified World Health Organization
PPCM: peripartum cardiomyopathy
TTE: transthoracic echocardiogram
